# Molecular Assessment of Neuroregenerative Response in the Pudendal Nerve: A Useful Tool in Regenerative Urology

**Published:** 2016-02-05

**Authors:** Bradley C. Gill, Dan Li Lin, Brian M. Balog, Charuspong Dissaranan, Hai-Hong Jiang, Margot S. Damaser

**Affiliations:** 1 Department of Urology, Glickman Urological and Kidney Institute, Cleveland Clinic, Cleveland, OH; 2 Cleveland Clinic Lerner College of Medicine, Education Institute, Cleveland Clinic, Cleveland, OH; 3 Department of Biomedical Engineering, Lerner Research Institute, Cleveland Clinic, Cleveland, OH; 4 Advanced Platform Technology Center, Louis Stokes Cleveland Veterans Affairs Medical Center, Cleveland, OH

**Keywords:** Pudendal Nerve, Neurogenic, Nerve Regeneration, Beta-Tubulin, Onuf’s Nucleus

## Abstract

**Aims:**

Assessing pudendal nerve neuroregenerative response provides valuable insight into injuries and regenerative treatments related to urinary incontinence. This project developed and validated a cost-effective, expedient, and adoptable method of assessing pudendal nerve neuroregenerative response.

**Methods:**

Sprague Dawley rats underwent unilateral pudendal nerve crush prior to spinal cord harvest and laser microdissection for separate collection of the injured and uninjured Onuf’s nuclei (pudendal motor neuron cell bodies). Commercially available kits were used to extract and isolate RNA, as well as reverse transcribe and amplify cDNA from cells. Utilizing standard quantitative polymerase chain reaction (Q-PCR), expression of β_II_-Tubulin, a cytoskeletal protein indicative of nerve growth and neuroregenerative response, was determined in the injured side relative to the uninjured side 1 week after injury.

**Results:**

Injury upregulated β_II_-Tubulin 2.36±0.46 times via Q-PCR, which was not significantly (p=0.508) different from the 2.49±0.38 times increase noted with in-situ hybridization previously. Starting with tissue collection, results are available within 1 day using PCR, while in-situ hybridization requires 4-weeks.

**Conclusions:**

An easily adoptable PCR-based method of assessing the neuroregenerative response of the pudendal nerve successfully reproduced results obtained with a previous radioisotope-based in-situ hybridization technique.

## INTRODUCTION

Continence is maintained by a complex system consisting of neurologic, muscular, and anatomic components.^1^ Current treatments target a number of these aspects when they have failed or become dysfunctional and urinary incontinence presents. These include implantable slings and reconstructive surgeries, neuromodulation via pharmacotherapy or electrical stimulation, injectable bulking agents orimplantable sphincters, as well as behavioral modifications. However, no current treatments address denervation of the urethral sphincter, espite innervation being essential to the continence reflex.^2^

As regenerative medicine continues to evolve, new treatments for the aforementioned etiologies of incontinence are being identified and studied. Stem cells have demonstrated promise in both restoring the anatomical support and functional sphincteric muscle involved in continence.^3^ Similarly, the administration of cytokines related to stem cells have also shown potential for incontinence treatment.^4–6^ With regard to denervation injury, neuroregenerative treatment of the pudendal nerve has also shown benefit for nerve regeneration and recovery from incontinence.^7,8^ The differing mechanisms and targets of these treatments necessitate unique studies to evaluate the efficacy and action of each.

Functional measures quantify degrees of incontinence and electrophysiological recordings show levels of sphincteric and neural activity.^9^ They provide insight into overall treatment outcomes but lack insight into the mechanisms at work. Likewise, routine histologic and cytologic studies can determine tissue recovery, while anatomic dissections may reveal the effects of surgical and injectable interventions.^10^ However, only specialized and complex neuroanatomical evaluations or tedious and time-consuming radioisotope in-situ hybridization methods provide insight into the effects of neuroregenerative treatments.^11–13^ As such, this project aimed to develop and validate a simple, efficient, and precise method for assessing the neuroregenerative response of the pudendal nerve without the need for radioactive reagents.

## MATERIALS AND METHODS

### Pudendal Nerve Injury

All experiments were conducted according to protocols approved by the local institutional animal care and use committee (IACUC). A total of 5 female, virgin, Sprague Dawley rats (225–250 g bodyweight) underwent unilateral pudendal nerve crush as previously described.^14^ Briefly, 100 mg/kg Ketamine and 10 mg/kg Xylazine intraperitoneal anesthesia was given, and a dorsal midline incision over the lumbar spine was used to gain access to the ischiorectal fossa through the lumbodorsal fascia. The posterior iliac crest was then lateralized to visualize the pudendal nerve, and a retractor placed to facilitate gentle dissection of the nerve and its isolation from the fascia. A Castro-Viejo needleholder was clamped twice, sequentially, across the entire pudendal neurovascular bundle for 30 seconds each time. The lumbodorsal fascia was closed with 3-0 silk suture through the gluteus superficialis while the skin was closed with a 3-0 polyglactin suture. Post-operative analgesia consisted of buprenorphine immediately upon anesthesia recovery and every 12 hours thereafter for 36 hours.

### Gross Dissection and Tissue Sectioning

**Figure 1:**
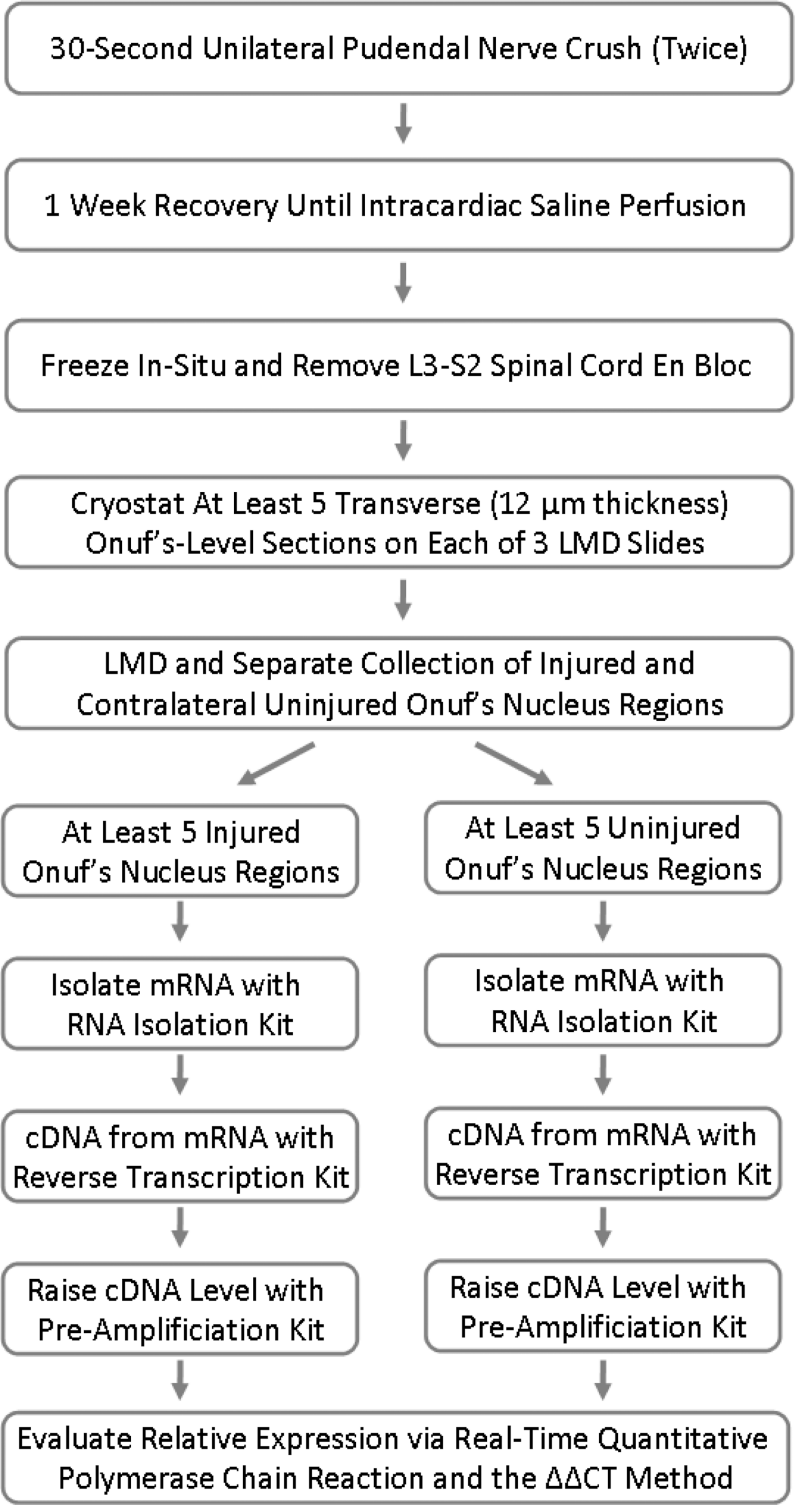
Overview of the PCR method for assessing neuroregenerative response in the pudendal nerve.

Tissue collection was performed as in prior studies.^15^ Specifically, 7-days after the unilateral pudendal nerve crush, animals were anesthetized as above and underwent intracardiac perfusion of heparinized phosphate-buffered normal saline. Upon satisfactory washout, as indicated by liver pallor, a midline dorsal laminectomy was performed. After exposing the spinal cord dorsally and laterally, liquid nitrogen was used to freeze the tissue in-situ [Figure 1]. The L3-S2 levels were sharply transected and the frozen segment of spinal cord was removed, placed into a pre-cooled cryotube, and stored in liquid nitrogen until cryostat sectioned.

**Figure 2:**
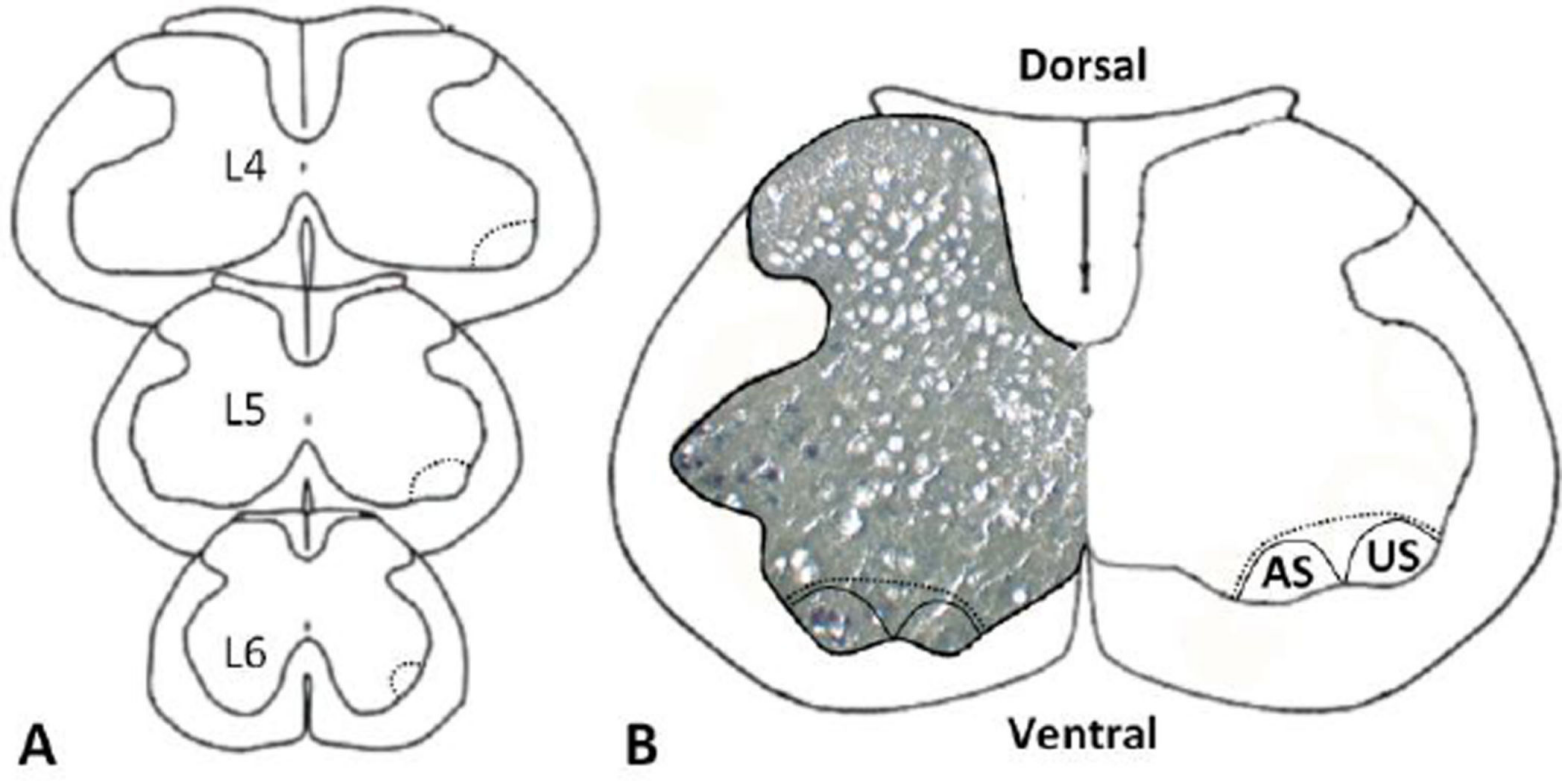
Schematic showing the rat L4–L6 spinal levels (A) near Onuf’s nucleus and a photographic overlay (B) of an L5 spinal cord section, depicting the separation of Onuf’s nucleus into the two distinct regions for the anal sphincter (AS) and urethral sphincter (US), which is the dorsolateral region. Neuronal cell bodies, stained blue-purple with thionin, are visualized as darkened spots on the grey-brown background staining.

Spinal cords were embedded in Tissue-Tek Optimum Cutting Temperature Compound (Sakura Finetek, Alphen aan den Rijn, The Netherlands) and placed on pre-cooled cryostat mounts. Serial transverse sections (12 µm thickness) were cut with intermittent samples collected on a glass slides, stained with thionin, and examined under light microscope until the L4/L5 region was identified, as modified from previous methods.^15^ Upon reaching the L4/L5 region, more frequent samples were analyzed with careful attention paid to the ventral horn and identification of Onuf’s nucleus, which contains the motoneurons of the anal and urethral sphincters, separated into 2 distinct regions, known as the dorsomedial and dorsolateral nuclei, respectively [Figure 2]. Upon noting distinct urethral sphincter nuclei, a minimum of 8 sections were placed onto each of 3 PET membrane laser microdissection (LMD) slides (PET Membrane LMD Slides, Leica Microsystems, Wetzlar, Germany) and stored at −80 °C until dissection.

### Tissue Fixation and Microscopic Dissection

**Figure 3:**
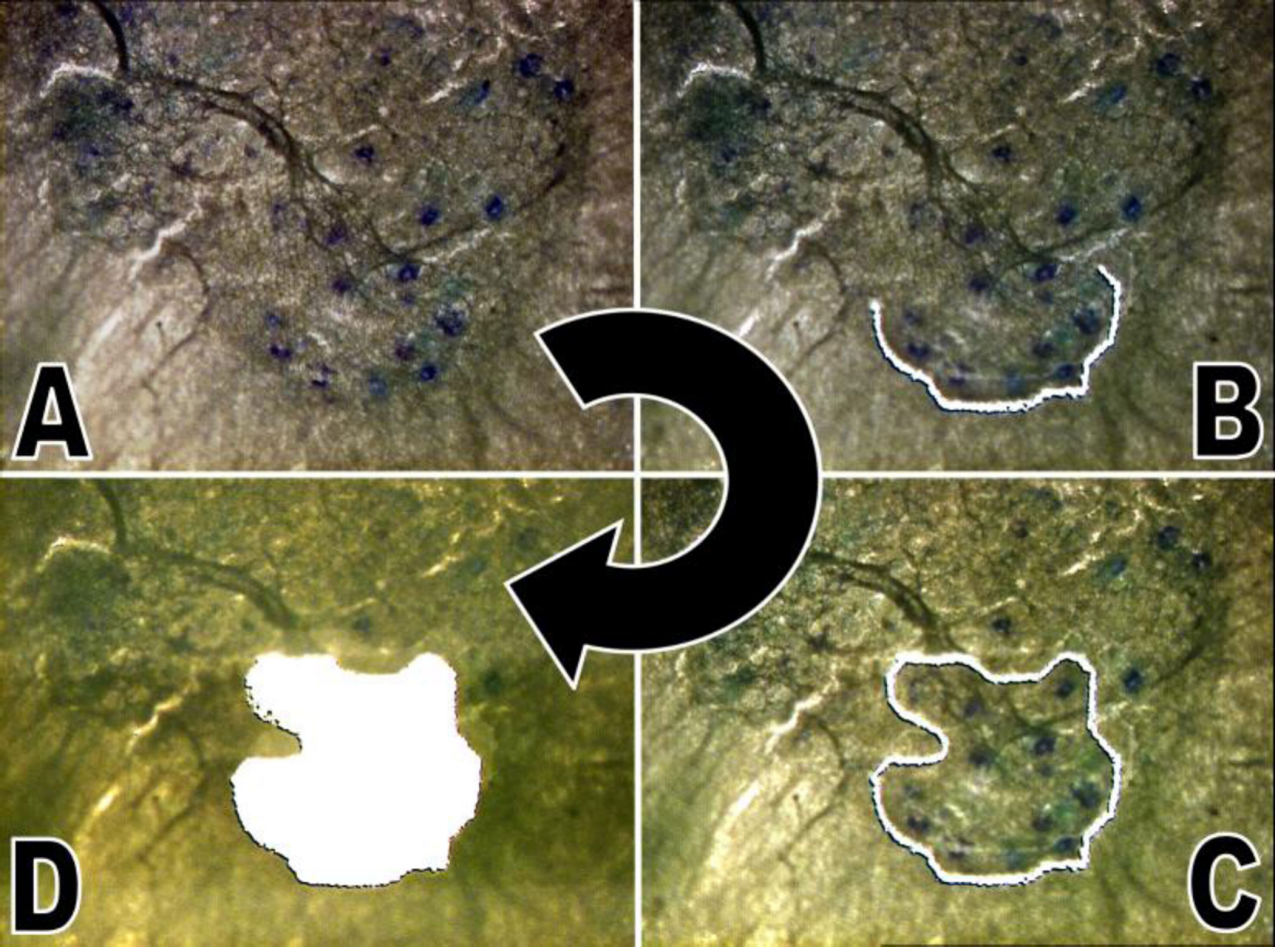
Neuronal cell bodies, stained blue-purple with thionin, are visualized as darkened spots (A) on the grey-brown background staining. Photographic sequence, progressing clockwise from the top left panel that depicts isolation (B and C) and dissection (D) of the urethral sphincter region of Onuf’s nucleus using laser microdissection. All images are 20× magnification.

Individually, slides were given 30 seconds to warm to room temperature, stained with thionin, dehydrated with ethanol, and fixed with xylene, as done previously.^11,15^ Specimens were dried for 3 minutes before being microdissected. A laser microdissection system (ASLMD, Leica Microsystems) was used to isolate and collect the cells of the dorsolateral region of Onuf’s nucleus, which contains the cell bodies of the motoneurons innervating the urethral sphincter via the pudendal nerve [Figure 3]. An RNase-free microcentrifuge cap filled with 40 µl of cell lysis solution (RNAqueous-Micro, Applied Biosystems, Foster City, California), which inhibits RNase activity, was used to collect the dissectate. A minimum of 5 cell clusters from the dorsolateral region of Onuf’s Nucleus, each from a different spinal cord section, were collected into separate microcentrifuge tubes from the injured and uninjured sides, respectively [Figure 1]. After collecting all samples from 1 animal, the microcentrifuge tubes were spun down at 10,000 RPM for 30 seconds and the caps rinsed twice with 30 µl of lysis solution and a subsequent spin down. Samples were stored at −20 °C until processed further.

### Nucleic Acid Isolation and Polymerase Chain Reaction (PCR)

Microcentrifuge tubes containing unilateral pudendal nerve cell bodies in lysis solution were thawed on ice prior to being processed with a commercially available ribonucleic acid (RNA) isolation kit (RNAqueous®-Micro, Applied Biosystems) containing a deoxyribonucleic acid (DNA) removal phase. Isolated RNA was utilized as a template for complementary DNA (cDNA) production using a reverse transcription kit (High Capacity cDNA Reverse Transcription Kit, Applied Biosystems) that is commercially available. Due to the small amount of genetic material being analyzed, the target cDNA concentration was increased by 14 polymerase chain reaction (PCR) cycles using a commercially available non-biasing pre-amplification PCR substrate (TaqMan® PreAmp Master Mix, Applied Biosystems) combined with a gene-spicific probe as done elsewhere when using LMD specimens.^16^ The probe targeted β_II_-Tubulin (TaqMan® Gene Expression Assay - Rn01435557_g1, Applied Biosystems), a cytoskeletal protein [Rat, Tubb2c Gene] upregulated during neuronal growth in both development and regeneration.^17,18^ After concentration, a portion of cDNA was taken and diluted with quadruple its volume of ultrapure, RNase-free water and the remainder stored at −20 °C for future use.

The relative expression of β_II_-Tubulin is indicative of the neuroregenerative response of the nerve and was assessed using real-time quantitative PCR (Q-PCR) performed with the same β_II_-Tubulin probe. Results were normalized to expression of 18S rRNA (TaqMan® Gene Expression Assay - Rn03928990_g1, Applied Biosystems), a structural RNA [Eukaryote, Structural Ribosomal RNA] that serves as a component of the 40S ribosomal subunit and is widely used as a Q-PCR endogenous control and has also been shown to have low expression variability in neuroregnerative studies.^19–21^ Both probes were combined with a high-efficiency PCR substrate (TaqMan® Gene Expression Master Mix, Applied Biosystems) designed to facilitate duplex PCR where two probes are simultaneously amplified and detected based. The Q-PCR reaction was run using the StepOne™ (StepOne™ Real-Time PCR System, Applied Biosystems) instrument with FAM™ and VIC® fluorescent labels designating the β_II_-Tubulin and 18S probes, respectively.

### Data Analysis and Statistical Methods

Relative expression of β_II_-Tubulin in injured and non-injured pudendal nerve cell bodies was calculated using the ΔΔCT method with 18S expression as the endogenous control accounting for differing amounts of genetic material.^22^ Automated threshold determination provided by the StepOne™ Software (StepOneTM Software v2.1, Applied Biosystems) was used to determine threshold cycle (C_T_) for each sample. Automatic threshold assignments for both probes were visually confirmed as being shortly after initiation of, and within, the exponential amplification phase. Normalized expression of β_II_-Tubulin in injured pudendal nerve cell bodies was calculated relative to that of the non-injured contralateral side in each rat. All values are expressed as mean ± standard error of the mean. A single-sample t-test in JMP (JMP 9, SAS Institute Inc, Cary, North Carolina) was used to compare the mean upregulation of β_II_-Tubulin expression to that previously obtained via in-situ hybridization with p < 0.05 indicating statistical significance.^15^

## RESULTS

**Figure 4:**
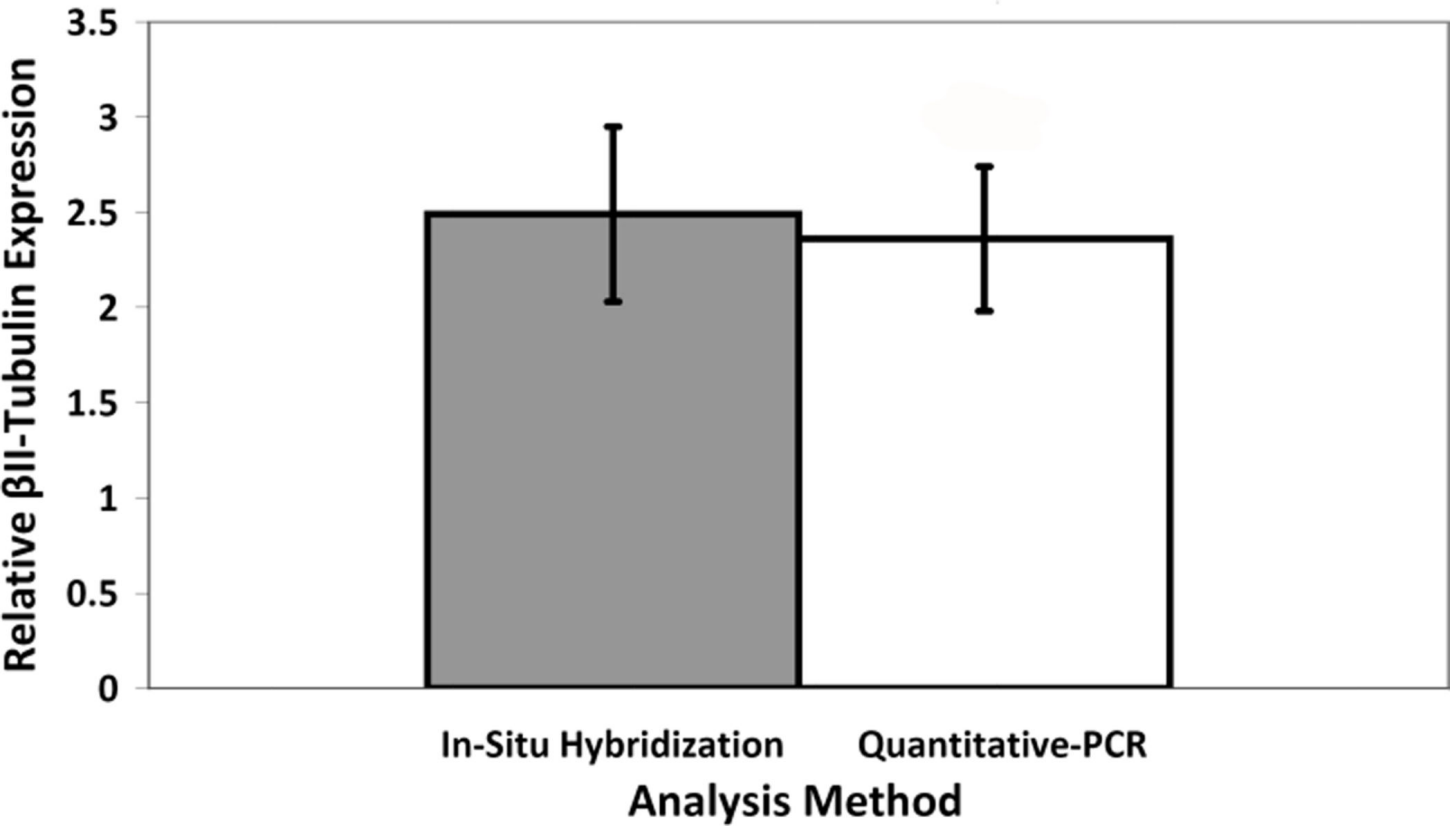
Comparison of a PCR-based method to the use of in-situ hybridization to assess the neuroregenerative response of the pudendal nerve 7 days after nerve crush injury, as signified by an increase in expression of the cytoskeletal protein β_II_-Tubulin relative to the expression levels in uninjured pudendal nerve cell bodies. No statistically significant difference in measured upregulation was detected. In situ hybridization data reprinted from Sakamoto, et al. 2000 with permission.

Following pudendal nerve injury, 1 rat was euthanized and excluded from analyses due to an adverse anesthetic reaction. The remaining 4 animals were successfully taken through the protocol to determine relative β_II_-Tubulin expression in the dorsolateral region of Onuf’s nucleus. The expression of β_II_-Tubulin was upregulated 2.36 ± 0.46 times 7 days after pudendal nerve crush compared to the contralateral uninjured side [Figure 4]. This was not significantly (p = 0.508) different from the 2.49 ± 0.38 fold increased expression detected via in-situ hybridization in previous work.^15^

## DISCUSSION

The field of regenerative medicine holds a number of possibilities for incontinence and voiding dysfunction. Already, stem cell treatments have been studied for use in restoring the structural integrity of the urogential organs and functional bulk of the urethral sphincter.^4,23–25^ A number of models exist for modeling sphincteric deficiency and its treatment, including recently published work on a pudendal nerve transection model, as well as the effects of insulin-like growth factor 1 (IGF-1) on recovery, respectively.^26,27^ Additionally, both electrical stimulation and the administration of neurotrophins, cytokines produced to facilitate nerve recovery, have improved functional aspects of the continence mechanism in models of post-partum incontinence.^7,8^ However, little insight into the effects of these treatments on neuroregenreation exists. As such, a method to assess the neuroregenerative response of the pudendal nerve could benefit research and development of novel treatments.

Axonal growth, either developmentally or during nerve regeneration, is facilitated by neuronal sprouting or outgrowth, which occurs via the production and extension of new cytoskeletal proteins.^11–13,17,28^ Measuring the expression of the genes for these structural proteins provides a method of determining if and how strong a neuroregenerative response has been mounted after nerve injury.^18,29^ Therefore, β_II_-Tubulin expression levels were used to assess the neuroregenerative response of the pudendal nerve in this project, as done previously.^15^

Prior measurement of the neuroregenerative response in the pudendal nerve utilized in-situ hybridization with radioisotopes to detect β_II_-Tubulin expression.^15^ This method required a 4-week period of film exposure to the radioisotope labeled cDNA, which was followed by the tedious process of counting microscopic grains overlying pudendal nerve cell bodies to quantify β_II_-Tubulin expression levels. Despite the opportunity for human error and high variability, the methodology was considered standard for assessing the neuroregenerative response, but remained costly in terms of time and labor. Often, it was only discovered after the 4-week incubation period that the results were unusable because of RNase contamination or other reasons. In addition, the use of radioisotopes involved additional risks to personnel as well as incurred extra costs due to necessary regulatory compliance.

In contrast to in-situ hybridization, a PCR-based methodology to assess neuroregeneration, such as the one described and validated here, can produce results from collected tissues in only 1 day. In addition, with the collection of the entire dorsolateral region of Onuf’s nucleus and automated measurements taken by Q-PCR machines and software, the influence of human error on results is reduced with this technique. Reliability, repeatability, and efficiency are also facilitated by numerous ready-made reagents, which greatly simplify and streamline the process. Furthermore, PCR does not require the use of radioisotopes, reducing the risk to researchers and time spent addressing regulatory requirements. However, despite these benefits, unlike in-situ hybridization, any PCR process is highly sensitive to contamination by other genetic material.

Limitations to the current study include the use of historical comparisons for validation, which was elected as a means of reducing the number of animals required for the study. With that, one benefit this provides is the use of a contralateral uninjured nerve for a normal control, as opposed to separate uninjured animals as done previously.^15^ Along with the use of historical controls for validation, the current study was performed using only Q-PCR for assessment of gene expression, as evidenced by mRNA levels. While this compares with the in situ hybridization of cDNA done previously, neither study has provided insight into the proteins produced with the gene upregulation by using either immunohistochemistry/fluorescence or a western blot analysis.^15^ Lastly, while the initial study provided insight into the baseline expression of β_II_-Tubulin, no such analysis is possible using the Q-PCR method as a relative assessment.^15^ However, this could be done if standard concentration samples were run on the PCR plates.

Nonetheless, similar protocols, described in detail, have been developed for the quantitative assessment of gene expression in LMD tissue samples.^19,30^ More recently, some of these have focused on levels of neurotrophins, molecules upregulated to stimulate neurodevelopment and neuroregeneration.^31–33^ The use of various genes, including 18S, have been validated as endogenous controls in LMD specimens.^19^ Much like the protocol described herein, LMD followed by TaqMan-based Q-PCR, with or without PCR pre-amplification of cDNA from isolated mRNA, has been utilized successfully to study other molecules.^16,34^ Thus, the current study, based upon historical comparisons, validates the use of this LMD and Q-PCR method to assess the pudendal nerve neuroregenerative response. Furthermore, based upon recent work demonstrating that retrograde neuronal labeling interferes with neuromuscular assessment, the serial-sectioning anatomical approach described herein makes this method applicable to specimens obtained after functional analyses.^35^

## CONCLUSIONS

Assessment of the neuroregenerative response of the pudendal nerve using a new PCR-based method successfully reproduces findings obtained using a more costly, time consuming, and hazardous in-situ hybridization process. Overall, the use of PCR in place of in-situ hybridization is more cost effective with regard to time and labor intensity, it can be performed in most laboratories, and the reagents it uses are safe and readily available. Considering these advantages, and the ability of this new technique to successfully replicate findings obtained with previous methods, the use of PCR to assess the neuroregenerative response of the pudendal nerve can serve as a useful adjunct to the many ongoing and future regenerative medicine projects related to incontinence and urology. In conclusion, this new molecular measurement of the pudendal nerve neuroregenerative response provides a useful addition to the armamentarium scientists and use to study the pathophysiology and treatment of urinary incontinence.
